# Factors influencing the routine immunization status of children aged 2-3 years in China

**DOI:** 10.1371/journal.pone.0206566

**Published:** 2018-10-31

**Authors:** Lei Cao, Jing-Shan Zheng, Ling-Sheng Cao, Jian Cui, Meng-Juan Duan, Qi-You Xiao

**Affiliations:** National Immunization Program, Chinese Center for Disease Control and Prevention, Beijing, China; University of Campania, ITALY

## Abstract

**Objectives:**

To examine the factors associated with the routine immunization status of children aged 2–3 years in China for gaining a better understanding of the Expanded Program on Immunization and to provide evidence for formulating specific strategies to guide the allocation of health resources.

**Methods:**

We analyzed data from 45095 children aged 2–3 years in the 2013 National Immunization Coverage Survey to identify the sociodemographic and provider-associated factors affecting the full immunization status of children. Univariate and multiple logistic regression analyses were performed.

**Results:**

The immunization rate for children aged 2–3 years ranged from 95.9% (diphtheria and tetanus toxoid with pertussis vaccine, 4^th^ dose) to 99.5% (Japanese encephalitis vaccine, 1^st^ dose) and was 93.1% for full immunization. In terms of sociodemographic factors, male children [adjusted OR (AOR): 1.115; 95% confidence interval(CI):1.016–1.222], minority children (AOR: 1.632; 95% CI: 1.457–1.828), children of fathers with less than high school education (AOR: 1.577; 95% CI: 1.195–2.081), those born at home (AOR: 4.655; 95% CI: 3.771–5.746), those who immigrated from an adjacent county (AOR: 2.006; 95% CI: 1.581–2.546), and those living in urban-rural fringe areas (AOR: 1.807; 95% CI: 1.475–2.214) or mountainous areas (AOR: 1.615; 95% CI: 1.437–1.814) had significantly increased odds of not being fully immunized. In terms of provider-associated factors, administration of vaccines at home (AOR: 2.311; 95% CI: 1.316–4.059), household reminders (AOR: 2.292; 95% CI: 1.884–2.789), and travel time to vaccination providers of >40 minutes (AOR: 1.622; 95% CI: 1.309–2.010) were negatively associated with immunization rates. In addition, compared to 3-year-old years, 2-year-old children (AOR: 1.201; 95% CI: 1.094–1.318) were less likely to be fully immunized.

**Conclusions:**

All included factors except maternal education level and distance from home to vaccination providers significantly affected immunization rates. Appropriate reminders and accessibility of immunization services played key roles in improving the immunization status. More attention to high-risk groups identified in this study may reduce the disparities in routine childhood immunization in China.

## Introduction

The Expanded Program on Immunization (EPI) is one of the most cost-effective public health interventions to protect children from vaccine-preventable diseases (VPDs) by delivering effective vaccines worldwide. The routine immunization program is a primary strategy of the EPI, which contributes to the reduction of both morbidity and mortality due to VPDs by vaccinating children aged 0–7 years and was initiated in 1978 with four vaccines in China [1]. After China’s central government decided to introduce new vaccines into the EPI at the end of 2007, including hepatitis A vaccine (HepA); epidemic meningococcal polysaccharide vaccine (MPV); measles, mumps, and rubella vaccine (MMR); measles and rubella vaccine; and Japanese encephalitis vaccine (JE), 22 doses of 11 EPI vaccines have been introduced into the routine immunization program for all children aged 0–7 years in China [2]. The Health Development Plan in China (2011–2015) proposed a goal of 90% for routine immunization coverage at the township level by 2015. Accordingly, a national immunization survey in China (CNIS) was conducted in 2013 to evaluate the completion of the goal and assess the immunization coverage level of children aged 2–3 years for 17 doses of eight vaccines administered before the age of 2 years [3].

Although China’s EPI has made remarkable progress in disease control in the past years, non-immunized children remain a public health concern [4,5]. A polio outbreak occurred in 2011 in the Xinjiang Uygur Autonomous Region of China after being polio-free for >10 years because of the introduction of wild poliovirus from neighboring Pakistan [6]; multiple measles outbreaks occurred in different geographical areas at different times [7,8], indicating that under-immunized populations remain an issue in China. To achieve the goal of disease control, understanding the factors that influence the full immunization of children holds practical significance. Therefore, we conducted this study using data from the 2013 CNIS to (1) identify factors that influence the immunization status of children in China aged 2–3 years using national representative samples, (2) improve our understanding of routine immunization in China, and (3) provide evidence for policy makers to improve immunization strategies going forward.

## Materials and methods

### Study setting and population

To date, the 2013 CNIS remains the most recent nationwide population-based household survey, which employed the methodology of the World Health Organization(WHO) and conducted strict quality control to ensure the validity of the survey. Children aged 2–3 years born between Sept. 1, 2009 and Aug. 31, 2011, randomly sampled from the 2013 CNIS were used in this study [3]. The CNIS used a structured questionnaire to collect data, which can be found in Supporting information (S1 Table). Data on the immunization status of children were obtained from parents (61.9%), grandparents (36.0%), or relatives (2.1%). Over 99% of the children had their immunization records kept at home, which were used for assessing their immunization status. If children were without immunization records, surveyors were required to visit the local health center to retrieve their immunization cards. Of note, children with neither immunization records nor cards were classified as non-immunized [4].

### Sample strategy

At the end of 2012, China had >16.5 million children born each year in 31 provinces and 40446 townships [9]. The 2013 CNIS was conducted in all 31 provinces, 963 counties and 1073 townships, accounting for 36.2% of the total counties and 2.6% of the total townships [4]. The survey used the lot quality assessment sampling method [10,11] to determine the sample size of children to ascertain whether the immunization coverage of EPI vaccines reached 90% at the township level; overall, at least 42 eligible children were required to be sampled in each sampled township.

The survey employed a two-stage sampling method to identify the townships and households. Firstly, we randomly sampled 29–35 townships based on the number of townships in each province after merging those townships with a population of <10,000 to meet sample size requirements. Five villages were then randomly sampled in each township, and the village with a township government was selected. Finally, 10 children from each village with a township government and 8 children from other selected villages were randomly chosen. The sample frame of households with eligible children was established based on the household registration, health record, or rural cooperative medical record. If a household had more than one eligible child, then the surveyor randomly sampled an eligible child to interview.

## Measures

### Dependent variables

The outcome variable in this study was the likelihood of children aged 2–3 years having missed at least 1 of 17 doses of eight required EPI vaccines (incomplete immunization), which included one dose of Bacillus Calmette–Guérin (BCG), three doses each of oral poliovirus vaccine (OPV) and hepatitis B vaccine (HepB), two doses each of measles-containing vaccine (MCV) and epidemic cerebrospinal meningitis group A polysaccharide vaccine (MPVA), four doses each of diphtheria and tetanus toxoid with pertussis vaccine (DTP), and one dose each of JE and HepA. If a child aged 2–3 years was immunized for all of the above 17 doses, then he/she was regarded as fully immunized. Immunization records at home and immunization cards at local health centers were used to identify the vaccination status of children. Children without records or cards were classified as non-immunized. Because routine immunization for JE has not been recommended in the province of Qinghai or in the Xizang and Xinjiang Autonomous Regions of China [3], children in these regions who had received 16 doses were considered to be fully vaccinated.

### Independent variables

There were nine sociodemographic variables, including age (2 vs. 3 years old), gender (male vs. female), ethnicity (Han vs. non-Han nationality), area (rural, urban, or urban-rural fringe) and terrain of residence (plain, mountainous, or hilly), parental education level (college, high school, or less than high school), birthplace (hospital, township health center, or home), and residence status of children (permanent resident, migrant child from an adjacent county, or migrant child from another province).

The four provider-associated variables were as follows: (i) immunization providers, or whether immunization was administered by doctors in community health centers (in urban areas), township health centers (in rural areas), village clinics (in remote rural areas), or at home (in extreme remote areas); (ii) type of vaccination reminder, which could consist of advanced booking of the next vaccination appointment at the time of immunization service, notifications of an upcoming immunization appointment, or household reminders (i.e., immunization record kept at home or self-recall); (iii) distance from home to vaccination providers, grouped as <5 km, 5–10 km, or > 10 km; (iv) and travel time from home to vaccination providers, grouped as: < 20 minutes, 20–40 minutes, or > 40 minutes.

### Statistical analysis

Data were analyzed using SPSS (SPSS Inc., Chicago, IL, USA) and statistical analyses were performed with the consideration of complex survey procedures. Univariate analyses were conducted to assess the relationship between immunization status and sociodemographic and provider-associated variables. Multiple logistic regression was utilized to determine the effect of the association. Variance inflation factors were calculated to exclude the possibility of multicollinearity between independent variables. None of these factors exceeded 10, which is the level suggested to be indicative of multicollinearity [12]. P-values of <0.05 were considered significant.

### Ethical Review

The Ethical Review Committee of Chinese Center for Disease Control and Prevention (China CDC) approved the study protocol of the CNIS. During the survey, participants were explained the study purpose and their right to privacy. Informed consent was verbally obtained before interviewing participants. The data used in the study were considered by China CDC’s Ethical Review Committee to be exempt from IRB review and were approved to be used by the China CDC.

## Results

### Recommended immunization schedule and vaccination coverage

Vaccination coverage in the 2013 CNIS and recommended immunization schedule for the EPI of China is shown in Table 1. In total, 45095 children aged 2–3 years were sampled. The immunization coverage of each reviewed vaccination in the 2013 CNIS was higher than 95%, ranging from 95.9% (DTP, 4^th^ dose) to 99.5% (JE, 1^st^ dose). The rate of fully immunized children aged 2–3 years was 93.1%.

**Table 1:** Recommended routine immunization schedule and percentage of children aged 2–3 years immunized in the 2013 CNIS, China.

EPI Vaccinations	Recommended Immunization Schedule	Number of Children Surveyed	Percentage of Children Immunized (%)
BCG	Birth	45095	99.0
OPV 3^rd^ dose	4 months	45095	99.1
HepB 3^rd^ dose	6 months	45095	98.9
MCV 2^nd^ dose	18–24 months	45095	97.4
DTP 4^th^ dose	18–24 months	45095	95.9
MPVA 2^nd^ dose	6–18 months	45095	96.5
JE 1^st^ dose	8 months	39749	99.5
HepA	18 months	45095	96.8
Fully immunized	–	45095	93.1

### Characteristics and univariate analysis

Table 2 summarizes the frequency of characteristics of children on sociodemographic and provider-associated factors. The results of univariate analysis on the association of exposure variables with incomplete immunization are shown with the crude odds ratio (OR) and confidence interval (CI) in Table 2. All variables except gender were significantly associated with an incomplete immunization status.

**Table 2:** Characteristics and crude odds ratios influencing the incomplete vaccination status of children aged 2–3 years in China.

Characteristics	Frequency	%	Crude OR	95%CI	P-value
**Age**					
3 years	20478	45.4	Ref.		
2 years	24617	54.6	1.128	1.048–1.215	0.001
**Gender**					
Female	21164	46.9	Ref.		
Male	23931	53.1	1.051	0.997–1.131	0.183
**Ethnicity**					
Han nationality	37090	82.2	Ref.		
Minority	8005	17.8	4.886	4.531–5.268	0.000
**Area of residence**					
Rural	30868	68.5	Ref.		0.000
Urban	11745	26.0	0.573	0.521–0.631	0.000
Urban-rural fringe area	2482	5.5	0.769	0.649–0.911	0.002
**Terrain**					
Plain	24310	53.9	Ref.		0.000
Mountain	13282	29.5	3.254	3.002–3.527	0.000
Hill	7503	16.6	1.209	1.072–1.363	0.002
**Maternal education level**					
College	5823	12.9	Ref.		0.000
Less than high school	31892	70.7	3.019	2.578–3.537	0.000
High school	7367	16.3	1.312	1.080–1.593	0.006
**Paternal education level**					
College	6376	14.2	Ref.		0.000
Less than high school	30747	68.2	3.125	2.682–3.641	0.000
High school	7935	17.6	1.374	1.141–1.656	0.001
**Birth place**					
Hospital	35368	78.4	Ref.		0.000
Health center	8480	18.8	1.363	1.239–1.500	0.000
Home	1247	2.8	19.110	16.941–21.557	0.000
**Resident status**					
Permanent resident	41796	92.9	Ref.		0.000
Migrant from an adjacent county	1133	2.5	1.347	1.095–1.657	0.005
Migrant from another province	2057	4.6	0.799	0.659–0.968	0.022
**Immunization provider**					
Community health center	11281	25.8	Ref.		0.000
Township health center	22565	51.5	1.712	1.534–1.909	0.000
Village clinic	8476	19.4	1.486	1.301–1.697	0.000
Administration of vaccines at home	1457	3.3	13.985	12.037–16.040	0.000
**Vaccination Reminder**					
Reservation	25873	59.8	Ref.		0.000
Notification	15972	36.9	1.536	1.414–1.668	0.000
Household reminders	1435	3.3	2.718	2.297–3.217	0.000
**Distance from home to provider**					
<5 km	37068	83.5	Ref.		0.000
5–10 km	4456	10.0	1.177	1.029–1.348	0.018
>10 km	2893	6.5	4.179	3.756–4.649	0.000
**Travel time to provider**					
<20 minutes	41810	94.9	Ref.		0.000
20–40 minutes	1831	4.2	1.307	1.157–1.476	0.000
>40 minutes	399	0.9	2.898	2.446–3.434	0.000

### Multiple logistic regression analysis

All variables were entered into a multiple logistic regression analysis, the results of which are shown in Table 3. Eleven of the 13 included variables (all except maternal education level and distance from home to vaccination providers) were significantly associated with an incomplete immunization status.

**Table 3:** Multiple logistic regression of factors associated with the incomplete immunization status of children aged 2–3 years in China.

Influential factors	Adjusted OR	95%CI	*P-value*
**Age group**			
3 years	Ref.		
2 years	1.201	1.094–1.318	0.000
**Gender**			
Female	Ref.		
Male	1.115	1.016–1.222	0.021
**Ethnicity**			
Han nationality	Ref.		
Minority	1.632	1.457–1.828	0.000
**Area of residence**			
Rural	Ref.		0.000
Urban	1.574	1.344–1.844	0.000
Urban-rural fringe area	1.807	1.475–2.214	0.000
**Terrain**			
Plain	Ref.		0.000
Mountain	1.615	1.437–1.814	0.000
Hill	1.337	1.165–1.534	0.000
**Paternal education level**			
College	Ref.		0.001
Less than high school	1.577	1.195–2.081	0.001
High school	1.265	0.966–1.658	0.087
**Birth place**			
Hospital	Ref.		0.000
Health center	1.337	1.196–1.494	0.000
Home	4.655	3.771–5.746	0.000
**Resident status**			
Permanent resident	Ref.		0.000
Migrant from an adjacent county	2.006	1.581–2.546	0.000
Migrant from another province	1.295	1.040–1.613	0.021
**Immunization provider**			
Community health center	Ref.		0.008
Township health center	1.129	0.961–1.325	0.139
Village clinic	1.017	0.835–1.239	0.868
Administration of vaccines at home	2.311	1.316–4.059	0.004
**Vaccination Reminder**			
Reservation	Ref.		0.000
Notification	1.127	1.011–1.256	0.031
Household reminders	2.292	1.884–2.789	0.000
**Travel time to provider**			
<20 minutes	Ref.		0.000
20–40 minutes	1.094	0.945–1.266	0.230
>40 minutes	1.622	1.309–2.010	0.000

## Discussion

The 2013 CNIS suggested that routine immunization coverage at the national level was high; however, it varied across provinces and type of vaccines because there were children with poor immunization status in some remote Midwestern provinces and Eastern urban areas [4]. Low income [13], low maternal education level [14,15] and areas of residence [16] are risk factors for lower child vaccination rates. Although research in China has suggested that minority children, children from central and western rural areas of China with less education and younger parents [17], and those from migrant families in urban areas [18–20] were at a higher risk of not being vaccinated, the determination of factors influencing the immunization status of children awaits a national-level study. In the present study, we used the 2013 CNIS data to determine factors associated with the full immunization status of children to elucidate the progress of EPI to date and provide evidence for the formulation of specific strategies to guide the allocation of health resources.

We found that sociodemographic factors, except for the maternal education level, affected routine vaccinations to differing degrees. In general, it is broadly accepted that maternal education level is an exogenous socioeconomic determinant that significantly protects child health and varies across countries and regions; better vaccine coverage is associated with higher maternal education levels [21,22]. Unlike previous studies, the present study found that paternal rather than maternal education levels were strongly correlated with immunization status in multivariate analyses, and consistent findings have been found in some areas of China [13,23] and the Western Pacific [24]. An explanation could be that higher paternal education levels are a proxy for higher family income and that family income positively affects the immunization status, although whether parental education level or income is more important in determining the health status of children remains debatable [25]. Surveys in the cities of Chengdu and Wenzhou found that family income is positively correlated with the immunization status of immigrant children [14,25]. Moreover, the probability of being fully immunized increased with paternal education levels, suggesting that improving paternal understanding on immunization improves the immunization status of children. This may represent an alternative to solely focusing on maternal health education.

In some Eastern countries such as India and China, compared with male children, female children are less likely to have access to immunization services [15,26,27]. However, after controlling for various other factors, we found that female children were slightly more likely to be fully immunized than male children in China. Similar studies have found that gender discrimination in terms of health status and general knowledge of the concept of “son preference” has been attenuated or even eliminated in recent years in China [28]. Overall, there are conflicting results on the effect of gender discrimination on immunization status, and results may depend on the research setting. Song [29] pointed out that in a family planning policy environment, the number of children that a family is permitted to have is subject to external restrictions. Son preference has changed to some extent, and gender discrimination may be reduced, particularly in families permitted to have only one child. However, we cannot dismiss gender differences in access to vaccinations because son preference continues to have a significant impact on children’s health in certain regions [28].

We also found that the age of children, area or terrains of residence, ethnicity, and residence status were significantly associated with vaccination status to varying degrees. The likelihood of being fully immunized appears to increase with age, most likely because older children have more opportunities to access immunization. Children living in urban-rural fringe areas were the least likely to be fully immunized than those living in other geographic locations, and children living in rural areas were more likely to be fully immunized than those living in urban areas. As a result of rapid urbanization, many immigrant populations have moved to urban-rural fringe areas, where a trend of lower immunization coverage and higher incidence of vaccine-preventable infectious diseases has been observed; these areas should be regarded as key areas for EPI management [20]. Moreover, among migrant children, those who migrated from other provinces seem to have had a better immunization status than those who migrated from adjacent counties. To our knowledge, similar results have not been reported elsewhere. This could be because the local EPI paid considerably more attention to children from other provinces than to those from local provinces when managing routine immunization management. In addition, migrant parents may have been unable to access information on immunization services in a timely manner due to their limited socioeconomic status and knowledge of immunization, particularly for new vaccines introduced in China after 2008 [30].

Children residing in mountainous areas, those born at home, and minority children had the greatest risk of being incompletely immunized. These results were consistent with those of previous surveys conducted in a mountainous area [31] and those conducted in the provinces of Sichuan [32] and Fujian [18]. Immunization services available to disadvantaged children in these areas were much weaker, lacked continuity, and were associated with a higher dropout rate because of transportation barriers, low socioeconomic status, limited access to health services, and inadequate immunization services [31].

Previous studies have shown that provider-associated characteristics are some of the most important risk factors for under-immunization [13,21,33], and information acquired from health-care workers was identified as one of the most influential factors for an adequate immunization among different population groups [34–36]. However, in China, several studies have primarily focused on parental satisfaction with immunization providers, rather than on other provider-associated factors, and on migrant children [19,26,37], rather than on all eligible children [14,30]. The present study found that vaccination providers, vaccination reminders, and travel time to vaccination providers were strongly associated with the immunization status among children. There were no significant differences in the immunization status among those vaccinated at community or township health centers compared to those vaccinated at village clinics. As mentioned by Linlin et al. [38], the administration of vaccines at home might be less effective than other vaccination settings in terms of achieving full immunization status. Consistent with findings from Nepal [39], travel time to vaccination providers was negatively associated with the immunization status, and travel time of <40 minutes may be preferable to avoid travel-related barriers to immunization in China.

Vaccination reminders are well-received and effective methods for improving immunization coverage [40]. In China, vaccination reminders, such as advanced appointment bookings or notifications, have long been included as routine methods for improving immunization coverage; however, few studies have examined the effectiveness of such reminders. The present study found that advanced appointment bookings might be more effective than other types of reminders, and the greatest risk of missing immunizations occurred when parents depended on nothing more than household reminders, such as immunization records or self-recall. A study in Beijing [41] further demonstrated that advanced appointment bookings set up via a mobile phone application are more effective and met parental requirements better than those manually set.

It is important to note the limitations of this study. First, this study involved the secondary analysis of data collected in the CNIS. As with all secondary datasets, data were limited by questions asked in the initial survey and responses may have been provided by a guardian who was not necessarily most familiar with the child’s health history. Secondly, the cross-sectional survey design utilized was less powerful than an analytical design would have been in terms of evaluating risk factors for incomplete immunization status. Finally, to simplify the operation of the investigation, we did not require the surveyor to collect the response rate, even it was high.

## Conclusions

This study was the first to assess a wide range of sociodemographic and provider-associated factors influencing the routine immunization status of children using nationally representative survey data in China. Further, this was a large population-based study with adequate power to detect small differences between groups, and 99% of immunization information based on the record minimizes the potential of information bias. Results revealed that all included sociodemographic and provider-associated factors, excluding only maternal education level and the distance from home to vaccination providers, significantly affected vaccination status among children. The probability of being fully immunized appears to increase with age, implying that children who are not able to be vaccinated on time were likely to be followed up and finish fully immunization. Furthermore, the study provided evidence that appropriate vaccination reminders and accessibility of immunization services play key roles in improving the routine vaccination coverage of children. Addressing these factors could contribute to identify at-risk children and geographic areas and could help develop corresponding strategies to reduce disparities in routine childhood immunization in China.

## Supporting information

S1 Table Household Survey Questionnaire in China. (DOCX)
