# Supplementary material for: Factors influencing the routine immunization status of children aged 2-3 years in China
Source: PLoS One. 2018 Oct 31;13(10):e0206566. doi: 10.1371/journal.pone.0206566 (PMC6209328; doi:10.1371/journal.pone.0206566)
Supplement: S1 Table — (DOCX) [file pone.0206566.s001.docx]

**Household Survey Questionnaire for Children**

Province County Township Village

Code for administrative division：

Code for Children:

| ***1. Characteristics of Children*** | |  | |
| --- | --- | --- | --- |
| a．area of residence | | 1 Urban 2 Urban-rural fringe area 3 Rural | |
| b. terrain | | 1 Mountain 2 Hill 3 Plain | |
| c. address | |  | |
| d. [respondent](javascript:;) | | 1 mother 2 father 3 Maternal grandparents 4 Other family member 5 Relatives | |
| e. maternal education | | 1 less than high school 2 high school 3 college or above | |
| f. paternal education | | 1 less than high school 2 high school 3 college or above | |
| g. name | |  | |
| h. gender | | 1male 2 female | |
| I. birth of date | | Year Month Day | |
| j. ethnicity | | 1 Han 2 Minority | |
| k. birth place | | 1 Hospital 2 Health center 3 Home | |
| l. resident status | | 1 Permanent resident 2 Migrant from an adjacent county 3 Migrant from another province | |
| m. immunization provider | | 1 Township health center 2 village clinic 3 Community health center 4 administration of vaccines at home | |
| n. distance from home to  provider | | 1 < 5km 2 5-10km 3 > 10km | |
| o. travel time to provider | | 1 < 20minutes 2 20-40minutes 3 >40minutes | |
|  | |  | |
| ***2. Immunization status*** | |  | |
| 1. immunization book | | 1 Yes 2 No | |
| b. immunization card | | 1 Yes 2 No | |
| c. BCG scar | | 1 Yes 2 No | |
| Vaccines | Dose | Immunization Date（yy/mm/dd） | Reason for immunization failure |
| BCG |  | / / |  |
| Hepatitis B | 1^st^ | / / |  |
|  | 2^nd^ | / / |  |
|  | 3^rd^ | / / |  |
| … | … | … | … |

Name of surveyor Date

Name of auditor Date
